# RNA Sequencing of *Arabidopsis thaliana* Seedlings after Non-Thermal Plasma-Seed Treatment Reveals Upregulation in Plant Stress and Defense Pathways

**DOI:** 10.3390/ijms23063070

**Published:** 2022-03-12

**Authors:** Alexandra Waskow, Anthony Guihur, Alan Howling, Ivo Furno

**Affiliations:** 1 Swiss Plasma Center (SPC), École Polytechnique Fédérale de Lausanne (EPFL), CH-1015 Lausanne, Switzerland; alan.howling@epfl.ch (A.H.); ivo.furno@epfl.ch (I.F.); 2 Department of Plant Molecular Biology, Faculty of Biology and Medicine, University of Lausanne (UNIL), CH-1015 Lausanne, Switzerland; anthony.guihur@gmail.com

**Keywords:** non-thermal plasma, RNA sequencing, *Arabidopsis thaliana*, oxidation-reduction, plant defense, secondary metabolism, glucosinolates, phenylpropanoids, wounding

## Abstract

Not all agricultural practices are sustainable; however, non-thermal plasma treatment of seeds may be an eco-friendly alternative to improve macroscopic plant growth parameters. Despite the numerous successful results of plasma-seed treatments reported in the literature, there is a large gap in our understanding of how non-thermal plasma treatments affect seeds, especially due to the plethora of physical, chemical, and biological variables. This study uses RNA sequencing to characterize the changes in gene transcription in *Arabidopsis thaliana* (L.) Heynh. seeds 6 days after exposure to surface dielectric barrier discharge plasma treatment. Here, we provide an overview of all pathways that are differentially expressed where few genes are upregulated and many genes are downregulated. Our results reveal that plasma treatment time is a parameter that can activate different pathways in plant defense. An 80 s treatment upregulates the glucosinolate pathway, a defense response to insects and herbivores to deter feeding, whereas a shorter treatment of 60 s upregulates the phenylpropanoid pathway, which reinforces the cell wall with lignin and produces antimicrobial compounds, a defense response to bacterial or fungal plant pathogens. It seems that plasma elicits a wounding response from the seed in addition to redox changes. This suggests that plasma treatment can be potentially applied in agriculture to protect plants against abiotic and biotic stresses without discharging residues into the environment.

## 1. Introduction

Currently, not every agricultural practice used to date is sustainable; however, non-thermal plasma treatment for seed treatment may be an alternative that produces no toxic residues, consumes little energy, has low penetration depth to avoid injuring cells while supporting seed development such as germination, and crop yield, as well as resistance to abiotic and biotic stresses when dosed adequately [1,2,3].

Cold, non-thermal plasma is an ionized gas, a complex mixture of energetic electrons, ions, UV, thermal radiation, electric field, and reactive oxygen and nitrogen species. Non-thermal plasma is unique; although the electrons are high energy at several thousand Kelvin, the overall gas temperature remains low. This is due to the efficient inelastic electron-molecule collisions, which create an exotic, high-temperature chemistry, and the inefficient elastic electron-molecule collisions, which avoid gas heating. Because of these properties, interesting biological applications of plasmas can be achieved. Dielectric barrier discharges (DBDs) at atmospheric pressure are often used, convenient for practical application in agriculture, and have an insulating dielectric layer that can be made of but not limited to glass, ceramic, plastic, or quartz. This layer is necessary to insulate the electrodes and avoid possible arcing due to the use of high voltages in the several kVs ranges. The plasma treatment time and duty cycle can be chosen to maintain a sufficiently low gas temperature, which is advantageous when working with heat-sensitive biological substrates such as seeds.

It has been shown that seed germination can be accelerated, or the plant properties can be altered where root length, shoot length, harvest mass or stress and disease resistance have increased after plasma treatment [4,5]. Despite the numerous successful results obtained in other studies, it remains elusive which plasma treatment parameters are required to affect plant growth parameters mainly because of the plethora of physical, chemical, and biological variables [6]. Therefore, the focus has recently shifted toward analyzing the gene transcription in seedlings and plants developing from plasma-treated seeds or even analyzing the methylation on genes or proteins using quantitative PCR (qPCR) or other high throughput methods, respectively [7,8,9,10].

Currently, the most common method for analyzing gene transcription in the plasma agriculture literature is qPCR, where specific genes of interest are targeted, but very few studies analyze genes in an unbiased manner using micro-arrays or RNA sequencing (RNA-seq) [11,12,13,14,15,16,17,18,19,20,21,22,23,24]. Furthermore, these findings are often only relevant to that plant and may not be applicable elsewhere to understand the molecular mechanisms of plasma-seed treatments. Therefore, it remains poorly understood which molecular mechanisms are involved in plasma-induced growth enhancement or disease and stress resistance. Although the plant choice is well justified based on local or global economic importance, there is often only one or a few studies of each plant type, which makes it difficult to compare the results and find commonalities. However, there are a few studies published recently using transcriptomics that provide a global overview of changes in gene expression after plasma treatment [25,26,27,28].

In this study, we use a surface DBD (SDBD) with two electrodes separated by the dielectric, and the plasma is formed in the gas at the edges of high-voltage patterned electrode to treat *Arabidopsis thaliana* (L.) Heynh. as a seed substrate. *A. thaliana* has its entire genome sequenced, and thus, unraveling the molecular mechanisms of the interactions with plasmas is more feasible. Since it is a plant model organism for the *Brassica* family, these findings could be potentially applied to agriculturally relevant crop plants. This study is among the first ones using RNA-seq in *A. thaliana* to characterize the changes in gene transcription in these seeds 6 days after SDBD plasma treatment using dry synthetic air. Here, we provide an overview of all pathways that are differentially expressed where a few genes are upregulated and many genes are downregulated. A hypothesis is proposed in the Discussion and Conclusions as to how plants could react to non-thermal plasma.

## 2. Results

### 2.1. Germination Rate of Plasma-Treated A. thaliana Seeds

**Figure 1:**
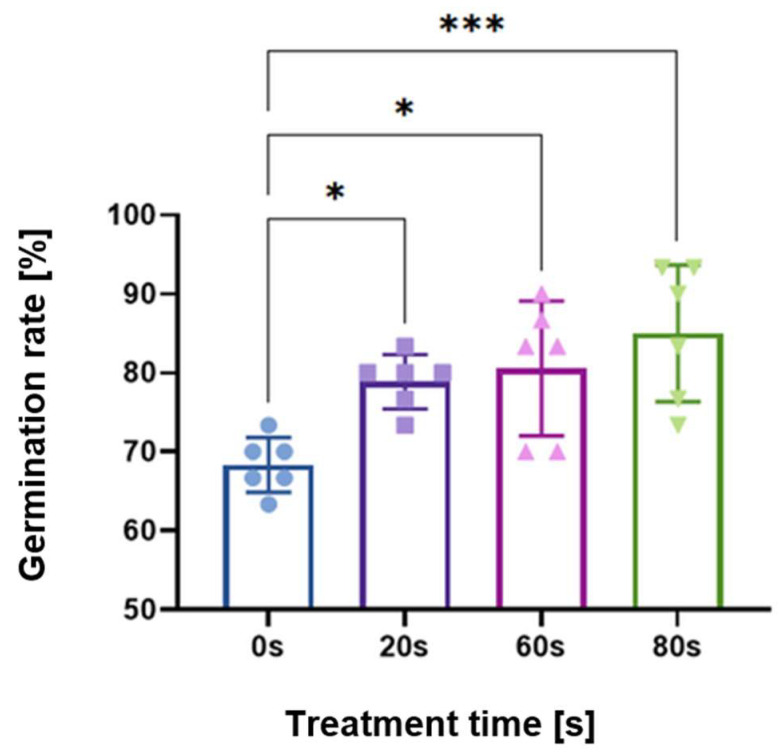
Germination rate results of plasma-treated *Arabidopsis thaliana* (L.) Heynh. seeds, demonstrating that 80 s had the strongest effect followed by the 60 s treatment. Control experiments, i.e., no plasma treatment, are indicated with 0 s. Shown as an average of triplicates performed twice independently for a total of 6 replicates. Asterisks denote statistical significance where * signifies *p* < 0.05; ** is *p* < 0.01; and *** is *p* < 0.001.

As shown in a previous study [29], germination rates were measured for parametric scans of treatment time, voltage, flow rate, gap distance between the plasma and seed, and frequency. Here, the operating parameters were either 60 or 80 s treatment time, 8 kV peak-to-peak; 2 L/min flow rate of dry synthetic air; seed substrate distance 3.7 mm; and 10 kHz frequency with a power on/off modulation at 500 Hz and 10% duty cycle, corresponding to a burst of 2 cycles per modulation period. The 10% duty cycle was used to avoid heat shock of the seeds. Figure 1 shows the germination rate of seeds measured after 48 h, compared to the control untreated seeds with no plasma exposure (indicated as 0 s in the figure).

As marked by the asterisks in Figure 1, scans of treatment time yielded statistically significant increases in the germination rate for 20, 60, and 80 s times. The longest plasma treatment time of 80 s had the highest statistical significance for the germination rate; therefore, this parameter set is the focus of this paper. However, data from 60 s are added and mentioned where appropriate.

### 2.2. Global RNA-Seq Analysis of Young Seedlings after Non-Thermal Plasma-Seed Treatment

To obtain gene expression profiles, RNA-seq data were generated from 6-day-old seedlings grown in continuous light. Each biological replicate is a pool of 30 seedlings grown in the same agar plate to reduce biological variability. An average of 33 million raw reads of 150 base pairs (bp) were produced for 80 s, while, after filtering steps, ~31 million clean reads (~94%) per library were retained (Table S1), and ~95.4% were mapped to the *A. thaliana* reference genome (Table S2). Similar values were obtained for 60 s.

**Figure 2:**
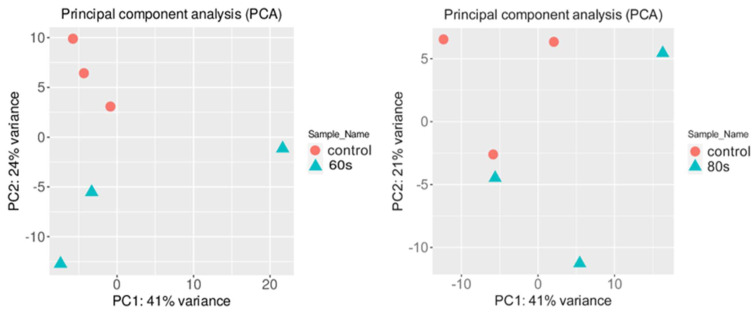
Principal component analysis (PCA) conducted on the normalized gene expression values of the 60 s (**left**) and 80 s (**right**) samples. X− and Y−axes show PC1 and PC2, respectively, with the amount of variance contained in each component, which is 41% and 24% for 60 s and 41% and 21% for 80 s, respectively. Each point in the plot represents a biological replicate, representing 30 seedlings, with a total of 6 biological replicates in the plot. Symbols of the same colors are replicates of the same experimental group where orange represents the control, which are untreated *A. thaliana* seeds grown into seedlings, and blue represents either 60 or 80 s plasma-treated *A. thaliana* seeds grown into seedlings.

To check if the RNA-seq data from different conditions were similar, a principal component analysis (PCA) was performed on the normalized gene expression values (Figure 2) [30]. The PCA analysis showed similar clustering among the replicates of each condition. One replicate of 80 s treatment slightly grouped with one control sample, which may be due to high biological variability, whereas two 60 s treatment replicates were more closely associated with the control samples. For the 60 s treatment, the first two principal components explained 65% of the total variance (41% by PC1 and 24% by PC2), whereas, for the 80 s treatment, 62% of the total variance was captured within the first two components (41% by PC1 and 21% by PC2). It is ideal to have most of the variance in the first PC vector; however, the data showed clear clusters between the samples and was used for further analysis. There was a total of 32,833 genes in 6 samples where 21,359 and 21,465 genes for 80 s and 60 s treatment, respectively, passed the selected threshold, which had more than 2 reads per biological replicate (see Methods).

**Figure 3:**
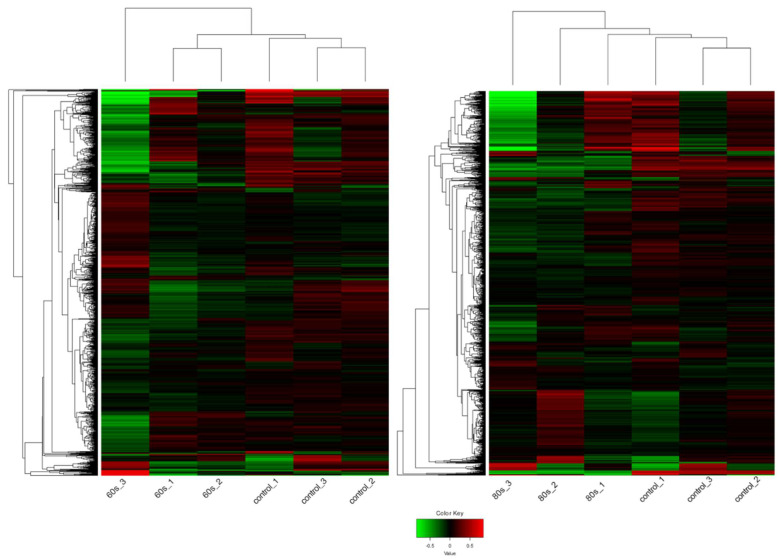
Heat map of the expression patterns (Z-scaled reads per kilobase of exon per million (RPKM) values) of the full transcriptome for 60 s (**left**) and 80 s (**right**). Hierarchical clustering of the relative expression profile of the top 2000 variable genes selected based on the lowest standard deviation using Euclidean distance. Individual samples are shown in columns and genes in rows. The upper axis shows the clusters of samples, and the left vertical axis shows clusters of genes. The color scale represents the relative expression of genes: green indicates low relative expression levels; red indicates high relative expression levels; black indicates zero (no change). The overall trend shows that genes are mainly downregulated after plasma exposure compared to the control, untreated samples.

**Figure 4:**
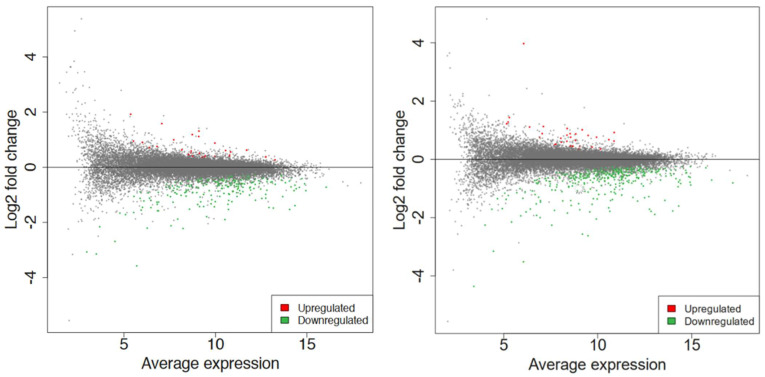
The MA plot shows the relationship between the average normalized expression on the x−axis and the significance of the differential expression test expressed as log2FC on the y−axis for each gene in the genome. It illustrates the number of DEGs for 60 s (**left**) and 80 s (**right**). Gray dots represent the genes that are not significantly differentially expressed, while red and green dots are the genes that are significantly up− and downregulated, respectively, based on their *p*−values (not shown here).

As shown in Figure 3, the hierarchical clustering demonstrates suitable agreement between the experimental groups where the control untreated samples clustered together as well as the 60 s or the 80 s treated samples despite the variation as can be viewed in the upper axis [31,32]. The variation could be due to the inherent biological variability between seeds, handling of the extraction, or different seed responses to the plasma treatment. Despite this, the quality of the RNA and mapping was very high (Figure S1, Tables S1 and S2). Compared to the 60 s treatment, the clustering was better in the 80 s treatment. Nevertheless, in both treatments, it is evident that there are more downregulated genes shown in green when treated with plasma compared to the control, which has more upregulated genes shown in red. This observation is displayed in an MA plot in Figure 4, where most genes are downregulated (shown in green), and only a few genes are upregulated (shown in red).

Differential expression analysis was conducted via DESeq2 v1.30.1 among all the conditions [33]. MA plot showed the extent of differential gene expression in response to plasma treatment. With a log2foldchange (FC) > 1 and false discovery rate (FDR) < 0.15, a total of 269 differentially expressed genes (DEGs) were obtained for 60 s treatment, where 27 genes were upregulated, and 242 were downregulated, whereas 422 DEGs were obtained for 80 s treatment, where 32 genes were upregulated, and 390 were downregulated, as shown in Figure 4.

**Figure 5:**
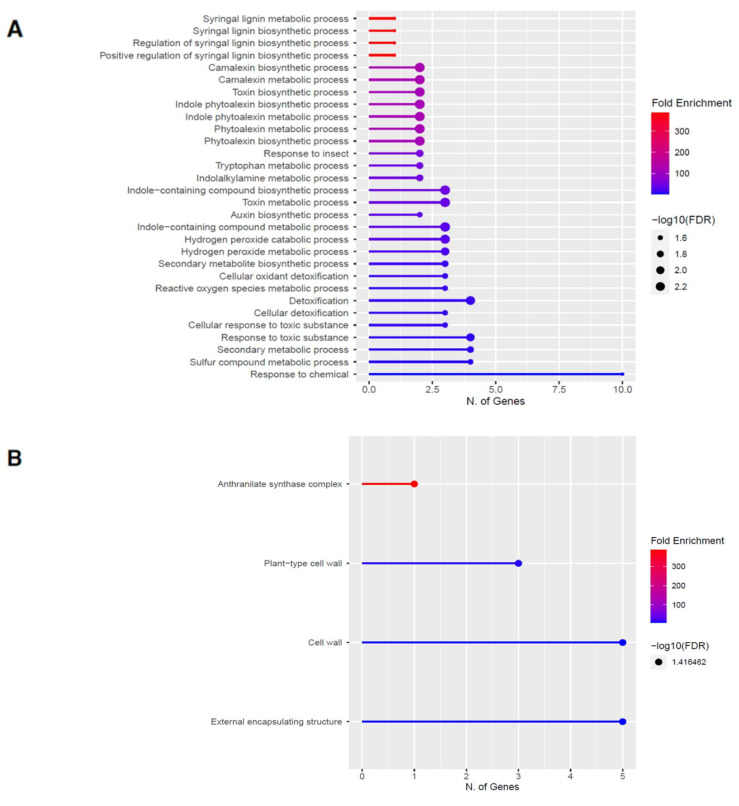

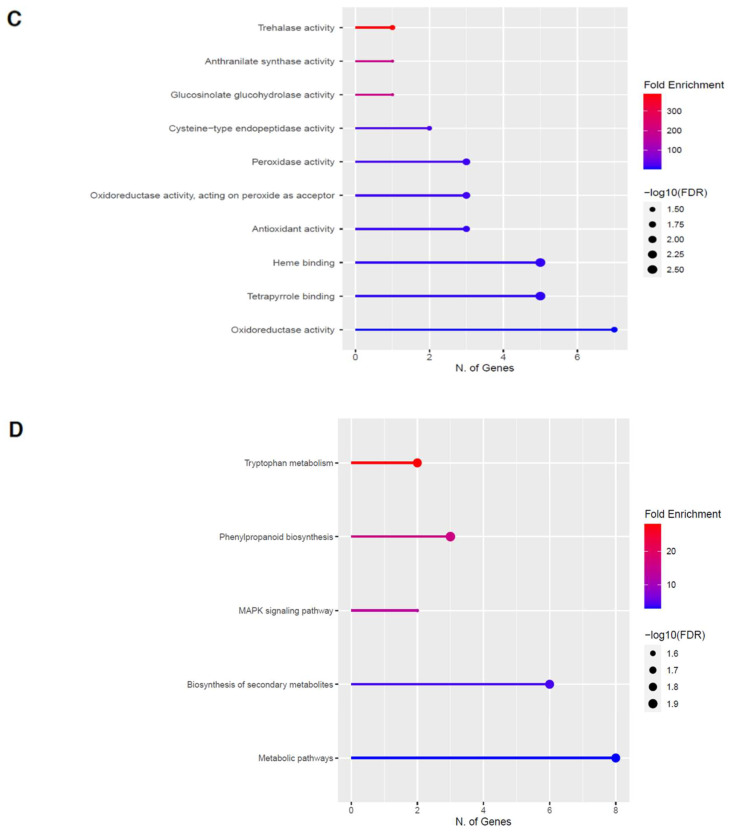
Gene enrichment analysis for upregulated genes after 60 s plasma treatment. Lollipop diagrams provide information about GO fold enrichment, significance (FDR in log10), and number of genes in each pathway. From top to bottom, GO categories are in the following order: (**A**) biological process, (**B**) cellular component, (**C**) molecular function, and (**D**) KEGG pathway.

**Figure 6:**
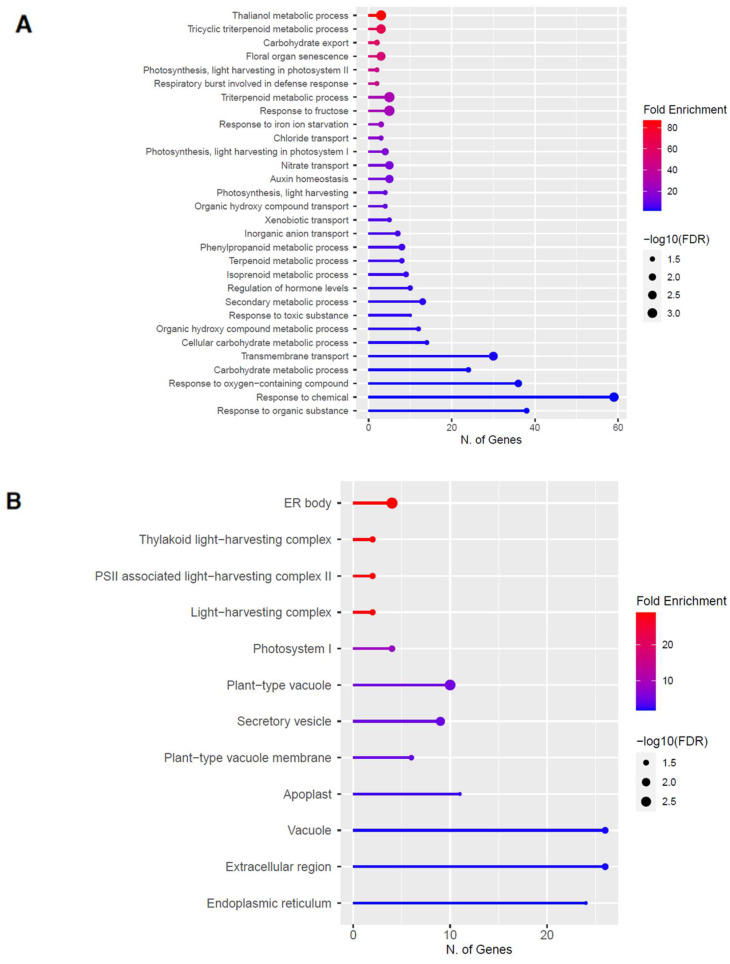

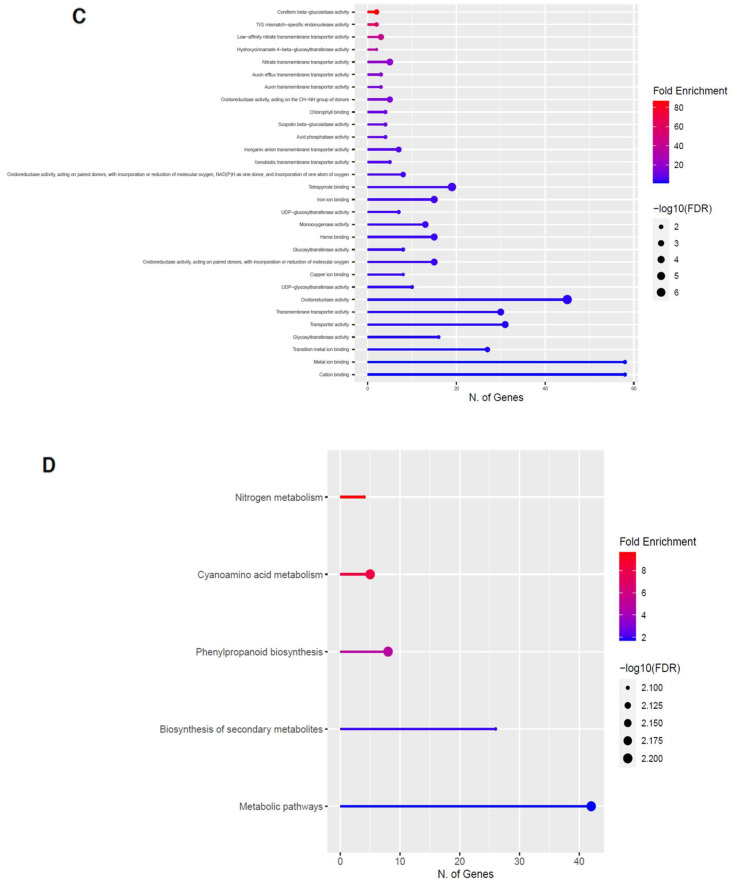
Gene enrichment analysis for downregulated genes after 60 s plasma treatment. Lollipop diagrams provide information about GO fold enrichment, significance (FDR in log10), and number of genes in each pathway. From top to bottom, GO categories are in the following order: (**A**) biological process, (**B**) cellular component, (**C**) molecular function, and (**D**) KEGG pathway.

**Figure 7:**
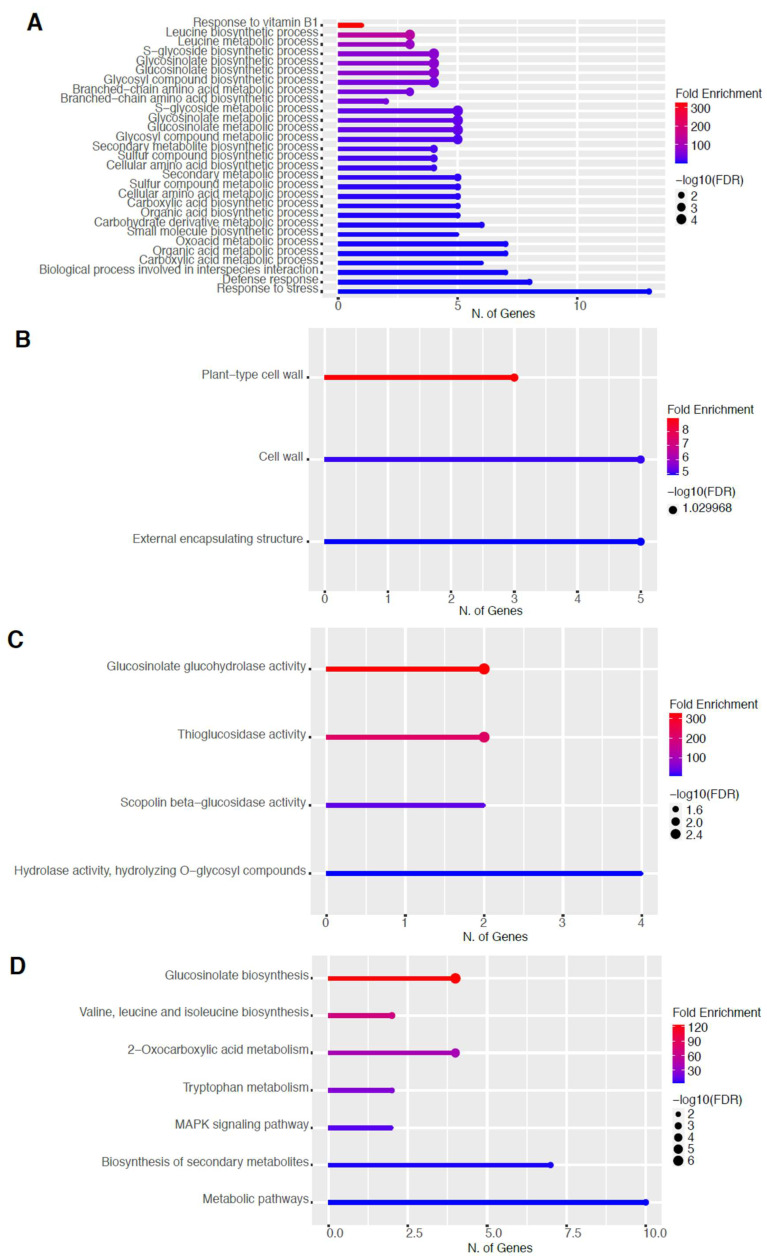
Gene enrichment analysis for upregulated genes after 80 s plasma treatment. Lollipop diagrams provide information about GO fold enrichment, significance (FDR in log10), and number of genes in each pathway. From top to bottom, GO categories are in the following order: (**A**) biological process, (**B**) cellular component, (**C**) molecular function, and (**D**) KEGG pathway.

**Figure 8:**
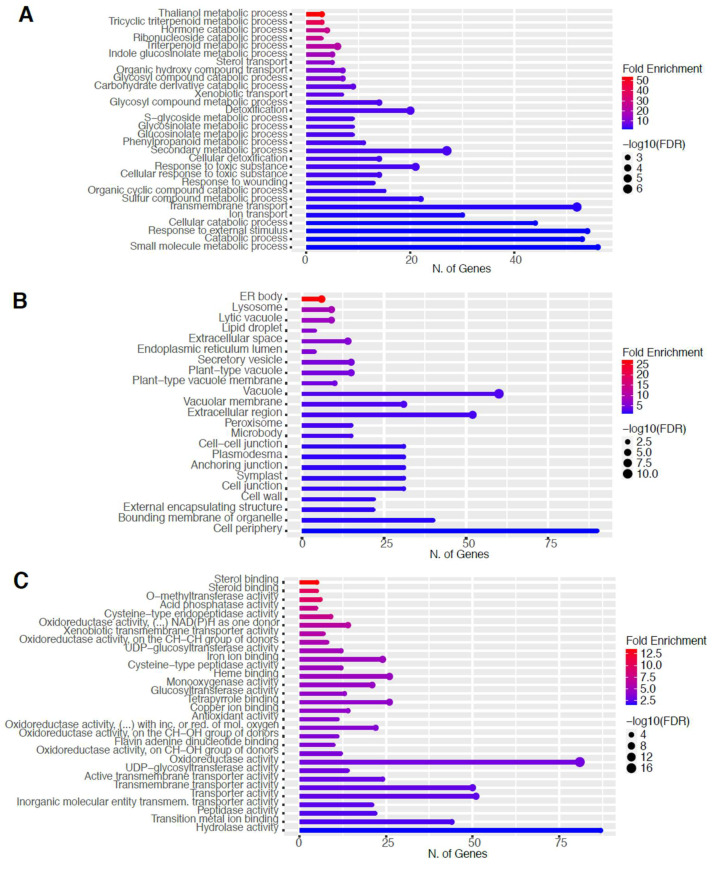

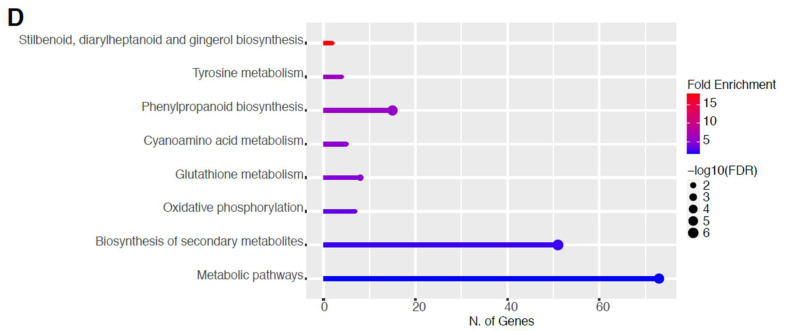
Gene enrichment analysis for downregulated genes after 80 s plasma treatment. Lollipop diagrams provide information about GO fold enrichment, significance (FDR in log10), and number of genes in each pathway. From top to bottom, GO categories are in the following order: (**A**) biological process, (**B**) cellular component, (**C**) molecular function, and (**D**) KEGG pathway.

To elucidate the potential biological functions of these DEGs, gene ontology (GO) analysis of specific groups of DEGs was performed using ShinyGO v0.66 software [34] with the biological process, cellular component, molecular function, and Kyoto Encyclopedia of Genes and Genomes (KEGG) pathways categories in Figure 5 and Figure 6 for 60 s plasma treatment and Figure 7 and Figure 8 for 80 s plasma treatment.

### 2.3. Upregulation of Genes in the Phenylpropanoid Pathway

The GO categories for biological process, cellular component, molecular function, and KEGG pathway enrichment (over-represented) are shown in Figure 5A–D, respectively, for the upregulated genes after 60 s plasma treatment. The lollipop diagram shows the fold enrichment and number of genes in the pathway, and the network map in the supplemental section shows the relation between the pathways (Figure S4). Overall, there is increased expression of genes involved in secondary metabolic pathways, specifically phenylpropanoids and metabolism of tryptophan, a precursor, as shown in Figure 5D. Specifically, lignin biosynthesis is highly upregulated as well as phytoalexin synthesis, whereas response to chemicals has the highest number of genes enriched, as shown in Figure 5A. Regarding the cellular component, anthrinilate synthase, an enzyme for tryptophan synthesis, as well as cell wall and related features, were upregulated, as shown in Figure 5B. The molecular functions that were upregulated in Figure 5C were enzymatic reactions related to trehalase activity and anthrinilate synthase.

### 2.4. Downregulation of Other Secondary Metabolic Pathways

The GO categories for biological process, cellular component, molecular function, and KEGG pathway enrichment are shown in Figure 6A–D, respectively, for the downregulated genes after 60 s plasma treatment. The lollipop diagram shows the fold enrichment and number of genes in the pathway, and the network map in the supplemental section shows the relation between the pathways (Figure S5). Overall, there is decreased expression of genes involved in generally other metabolic pathways, one of which is the phenylpropanoid pathway along with nitrogen metabolism, as shown in Figure 6D. In Figure 6A, the thalianol and triterpenoid pathways were the most downregulated, whereas the highest number of downregulated genes was observed in response to chemical or response to oxygen-containing compounds. In Figure 6B, there was a more diverse response from organelles after plasma treatment where many organelles, especially the ER body and photosynthetic apparatus, were downregulated. In Figure 6C, auxin transmembrane transport activity was among the most enriched molecular functions, whereas the highest number of downregulated genes were involved in cation binding.

### 2.5. Upregulation of Genes in the Glucosinolate Pathway

The GO categories for biological process, cellular component, molecular function, and KEGG pathway enrichment (over-represented) are shown in Figure 7A–D, respectively, for the upregulated genes after 80 s plasma treatment. The lollipop diagram shows the fold enrichment and number of genes in the pathway, and the network map in the supplemental section shows the relation between the pathways (Figure S8). Overall, there is increased expression of genes involved in secondary metabolic pathways, specifically glucosinolates and the precursors for their synthesis, such as valine, leucine, and tryptophan, as shown in Figure 7D. Specifically, the response of vitamin B1 is highly upregulated, whereas the defense and stress responses have the highest number of genes enriched, as shown in Figure 7A, all of which are interconnected. Regarding the cellular component, only the cell wall and related features were upregulated, which included several genes, as shown in Figure 7B. The molecular functions that were upregulated in Figure 7C were enzymatic reactions related to glucosinolates. All of these upregulated elements point to stress and defense responses being upregulated after 80 s plasma treatment.

### 2.6. Downregulation of Other Secondary Metabolic Pathways

The GO categories for biological process, cellular component, molecular function, and KEGG pathway enrichment are shown in Figure 8A–D, respectively, for the downregulated genes after 80 s plasma treatment. The lollipop diagram shows the fold enrichment and number of genes in the pathway, and the network map in the supplemental section shows the relation between the pathways (Figure S9). Overall, there is decreased expression of genes involved in secondary metabolic pathways, specifically phenylpropanoids, phenolic compounds such as gingerol and stilbenoid, tyrosine as a precursor of the phenylpropanoid pathway, and glutathione metabolism, as shown in Figure 8D. In Figure 8A, the thalianol and triterpenoid pathways were the most downregulated, whereas the highest number of downregulated genes were observed in catabolic processes. In Figure 8B, there was a more diverse response from organelles after plasma treatment where many organelles, especially the ER body, lysosome, and vacuoles, were downregulated. In Figure 8C, steroid and sterol binding were the most enriched, whereas the highest number of downregulated genes were involved in enzymatic processes.

## 3. Discussion and Conclusions

As stated previously, it is difficult to compare results across plasma-seed treatment studies due to the high number of variables, and if molecular analysis is performed, often specific genes are selected based on economic or health-related importance. In previous plasma agriculture studies, the genes that were targeted are related to germination, primary or secondary metabolisms, such as starch-degrading enzyme [11], drought-related resistance genes [12], antioxidant genes [13], pathogen resistance (PR) genes, and epigenetic regulation related genes [14,15], and plant-specific secondary metabolites with pharmacological uses [17,19].

The aim of our study was to look at the long-term memory effect of the plasma treatment by using 6-day-old seedlings rather than 1–2-day-old seeds because both the root and stem would have emerged from the seed, and this would ensure that there would be transcriptional changes that could be absent if the extraction is performed too soon; this was the case in the *Andrographis* study [25]. Moreover, it is common practice to use young seedlings because they are more sensitive to stress, and therefore, it would be possible to observe acute stress. A limitedtreatment time was chosen to avoid additional stresses such as heat stress, which induces a plethora of cellular effects [35,36,37,38]. Early germination was the macroscopic parameter used as an indicator of changes in the molecular biology, as shown in Figure 1, which is an effect observed in other studies [39].

For this first exploratory study, RNA-seq was used since it is an unbiased method that provides a global overview of all genes. DESeq2 was used for this paper since it is common practice and to balance stringency and flexibility [40]. We analyzed approximately 21,000 *A. thaliana* genes out of approximately 33,000 genes using RNA-seq (NCBI project number PRJNA800224) on plasma-treated seeds grown until and including the sixth day. We found 422 DEGs, with 32 upregulated and 390 downregulated (Figure 4), and suitable agreement between the biological replicates within the control and 80 s plasma-treated samples (Figure 3). For the 60 s plasma-treated samples, we found 269 DEGs, with 27 upregulated and 242 downregulated (Figure 3 and Figure 4).

### 3.1. Gene Enrichment as a Result of Short Non-Thermal Air Plasma Treatment

As shown in Figure 1, accelerated germination was observed, which is in line with the growth enhancement effects observed in other studies [4,41]. Growth enhancement is not the only type of plant response to plasma and also includes seed surface functionalization, seed decontamination, as well as stress anddefense response [1]. Our findings in Figure 5 and Figure 6 for 60 s and Figure 7 and Figure 8 for 80 s suggest that on a molecular level, this increased germination rate is mainly an outward expression of stimulated stress and defense response. However, this does not exclude other effects such as cell wall modifications, ion homeostasis, and modified plant microbiome interactions as a result of plasma treatment. Therefore, these will be briefly mentioned, followed by the main focus of our findings about the plant defense response.

Plasma is known to modify the seed surface directly, and therefore, this could explain the upregulation in cell wall cellular components seen in Figure 5B and Figure 7B. Bafoil and co-authors showed that the total activity of peroxidases increased in plasma-treated seeds, which were also enriched here, and peroxidases can trigger internal changes that subsequently externally modify the cell wall [42]. It is more likely that this change has a chemical basis since the plasma treatment here was indirect with a 3.7 mm plasma-seed gap distance, and thus, it is unlikely, although not impossible, that there was much interaction between the seeds and electrons, ions, or electric fields confined close to the electrodes.

Through seed surface modifications, it is also possible to alter ion homeostasis, which was indicated by downregulation in ion transport activity in Figure 8A. Previous studies have shown ion redistribution after plasma treatment in cations such as calcium, magnesium, or potassium, migrating into the interior of the seed or being enriched on the surface [43,44]. This, however, could also be interpreted by the seed as damage and, therefore, could be a symptom of a plant stress response.

It could be that through this stress response, plants modulate their own root activity, thereby altering their microbiome interactions. Plasma is very commonly known for its decontamination application in order to remove bacteria [45,46], but recent studies have demonstrated that plants treated with plasma modulate their relationship with the microbiome; root activity is modified, and nodulation is increased [8,9,47]. As shown in Figure 6A, our data revealed changes in the thalianol pathway, which could explain changes in plasma-treated plant root performance observed in other studies. This pathway is involved in root-specific metabolites to encourage plant-bacteria interactions and was shown to be important for shaping the *A. thaliana* root microbial community (Huang et al., 2019). Additionally, triterpenes were also downregulated (Figure 8A), and these compounds can influence metabolite exudation and therefore indirectly modulate the rhizobiome and root bacteria to have either growth-promoting or inhibitory effects [48].

Based on our gene enrichment analysis in Figure 7A,C,D, our main findings were an increased defense response, specifically of the glucosinolate pathway with an 80 s plasma treatment time several days after observing accelerated germination. The production of secondary metabolites involved in plant defense has been reported previously as well as changes in glutathione, an antioxidant involved in detoxification, particularly of reactive oxygen and nitrogen species during stress in living organisms [25,26,28]. Our data in Figure 5, Figure 6, Figure 7 and Figure 8 suggest that the plant may still be responding to the stress even 6 days after the initial treatment where a continuous cascade of programs has been triggered, which affects both primary and secondary metabolisms.

Primary metabolism is used for growth; however, it has the precursors or building blocks for secondary metabolites. These precursor compounds are mainly from the pentose phosphate (PP) pathway for the synthesis of phenolic compounds within the phenylpropanoid pathway or glucosinolates, whereas other intermediates from glycolysis can be used toward the mevalonic or methylerythritol 4-phosphate (MEP) pathway to produce terpenes and sterols [49]. Therefore, increased primary metabolism such as the production of precursors PEP, acetyl CoA, and 3-phosphoglycerate for secondary metabolites might result in the upregulation of organic acid pathways. Alternatively, branched-chain amino acids such as leucine or aromatic amino acids such as tryptophan or tyrosine, as shown in Figure 7A,D, Figure 8D, and Figure S4D, can be shuttled to produce these phenolic compounds or glucosinolates.

Glucosinolates are categorized into tryptophan-derived indole, tyrosine or phenylalanine-derived aromatic, or aliphatic glucosinolates. Biosynthesis of aliphatic glucosinolates starts out from alanine, valine, and leucine, with the most abundant group of aliphatic glucosinolates synthesized from methionine [50]. This would explain the upregulation of branched-chain amino acids as well as the increase in B1 (Figure 7A), a vitamin that is involved in amino acid synthesis, pentose phosphate, and TCA cycle and is being increasingly recognized and linked with plant defense [51,52]. Moreover, this complements the observed enrichment of oxoacid Figure 7A and Figure S6A), which is linked with generalist herbivory, possibly through glucosinolates [53,54].

How this stress signal is transduced based on the DEGs is largely due to mitogen-activated protein kinases (MAPK), as seen in Figure 5D and Figure 7D. There are multiple MAPK pathways that are involved in hormone signaling and trigger various stress responses due to other abiotic and biotic factors [55,56]. After the signal is perceived, the organelles that are the most affected by the plasma treatment are related to the cell wall, which was upregulated, and ER body, which was downregulated (Figure 5B, Figure 6B, Figure 7B, and Figure 8B).

It was unsurprising to find changes to the cell wall since it is the first point of contact between the seed and plasma. The effect on the cell wall could include reorganization to strengthen its defense against stress. However, it was anticipated that the lysosomes and peroxisomes would also be upregulated due to oxidized, damaged macromolecule degradation from exposure to plasma-derived RONS. In Figure 8B, the opposite was determined where lysosomes were downregulated. This might be because a perceived mild or moderate stress results in a transient upregulation, ranging from seconds to days. Since the mRNA was extracted at a later development stage, this could have been missed, and the cells could have adapted by then to the stress.

Many of the organelles were downregulated, especially the ER body, which was observed with both plasma treatment times (Figure 6B and Figure 8B). On the one hand, with the increase in glucosinolates, it could be expected that the ER body, which houses these compounds, would also be upregulated [57]. On the other hand, it could be that there are more than enough defense resources already available and stored in anticipation of the next attack, and therefore, the ER body downregulated its activity as a means to not compromise resources still required for growth.

### 3.2. Comparison with Other Transcriptomic Studies

To verify whether our interpretations are valid, the most relevant comparison can be made with the results available in another study using *A. thaliana* and, where appropriate, other transcriptomic studies [25,26,27,28]. A detailed comparison between our experimental setup and that of Cui and co-authors is shown in Table S3.

One of the main differences was in the materials of the plasma device as well as in the plasma setup, which used a continuous sinewave power supply with varying frequency, whereas ours used a 10% duty cycle. Their plasma chemistry may have differed where they measured atomic oxygen and NO with OES, whereas we used Fourier transform infrared spectroscopy (FTIR) and found that we operated mainly in ozone mode with a lower concentration of NO_x_, although NO was present within a few mm from the SDBD from preliminary laser-induced fluorescence (LIF) measurements [29]. There were greater differences in the seed handling. Cui and co-authors worked with sterilized seeds, whereas our conscious choice was to keep the native microbiome even though we were aware that plasma can interact with microorganisms and change the plant growth upon their removal. However, avoiding the rehydration of the seed with a sterilization protocol was a higher priority for this study than sterilizing the seed since this can change the metabolic activity, add additional stress to the plant, and thereby possibly change the final results. It is understandable that Cui et al. placed the seedlings in water to avoid dehydrating and thus stressing the seedlings, although it should be borne in mind that the presence of water can change the chemistry of the plasma considerably. Therefore, not only is the substrate different, seedling instead of seed, but the chemistry could also be different. Nevertheless, it is encouraging to see significant overlap between these studies because this promises some robustness in the experimental results, and this might be explained by using the same seed type, similar operating parameters for the plasma-seed treatment, and extraction time point [6]. The RNA extraction was performed 48 h after plasma treatment on 4-day-old seedlings, so, both studies extracted from 6-day-old seedlings, and the main difference was that plasma treatment was performed on either the seed or seedling. Both studies achieved changes in germination using a similar treatment time interval of around 1 min; however, stronger effects were observed in our study using 80 s. Heat was monitored and controlled in our study to better delineate the mechanisms by limiting additional stresses other than plasma. It has also been demonstrated that shorter treatment times, such as 1 min, are more effective than longer treatment times such as 3, 5, or 10 min.

Three studies, including ours, show a similar general pattern of predominantly downregulated genes [26,28]. However, this is not the case for other studies [25,27]. These differences may be due to physiological differences between different organisms but also to the plasma conditions or the selected RNA-seq approach. Cui and co-authors found enrichment in glutathione metabolism, MAPK signaling pathway, indole alkaloid biosynthesis, and plant-pathogen interaction pathway, which confirms the trends observed in our study. A study using sunflowers showed a shift toward the phenylpropanoid pathway rather than the glucosinolate, and judging by the short treatment time, it could be more similar to our results after 60 s treatment. Since we include two plasma treatment times, it is possible to propose a model based on the findings in this study where increasing the treatment time shifts the response of the plant from the phenylpropanoid pathway, which reinforces the cell wall with lignin and rapid response with phytoalexins or camalexin at 60 s (Figure 5 and Figure 6), toward glucosinolate production at 80 s (Figure 7 and Figure 8) as if the plant is protecting itself from an insect or herbivore attack. Therefore, depending on the intensity of the plasma, varying biological responses can be elicited from the plant.

Regarding secondary metabolism, Cui and co-authors found mostly a decrease in amino acids except for an increase in tyrosine and tryptophan after plasma treatment. In our study, it is clear that leucine, valine, and tyrosine were the key amino acid precursors for subsequent secondary metabolite production such as glucosinolates and phenylpropanoids, and therefore, it is in agreement with their findings. Although they had a high enrichment of glutathione-related processes, in our data set, it was not as pronounced as glucosinolates or phenylpropanoids. Glutathione is involved in the biosynthesis of glucosinolates, specifically specialized glutathione-S-transferases (and for phytoalexins) [54]. One explanation could be that glucosinolate production is supported, and since the treatment was performed on the seed surface, the RONS, whether plasma- or plant-derived, may have dissipated shortly after germination and at the point of extraction, detoxification was no longer necessary and therefore was downregulated; perhaps even reset at a new threshold with the activated stress response.

Triterponoids are also defense compounds; however, they were downregulated. To attempt to explain this, it may be a question of how to best use resources where the plant decides it is preferable to use glycolysis products toward the synthesis of specific phenylpropanoid compounds or glucosinolates, especially with a common precursor such as tryptophan (Figure 7D and Figure 8D). For example, stilbenoids were downregulated and are classified as phytoalexins and phenolic compounds [58]. Therefore, resources might have been shuttled toward glucosinolates instead sinceit has been shown that the glucosinolate pathway can limit phenylpropanoid production [59]. Alternatively, perhaps it is also faster to produce phenylpropanoids than glucosinolates, or it is launched as an earlier line of defense.

Although all other transcriptomic studies reference plant hormones as an important reason for the macroscopic changes, the only plant hormone detected here was auxin [25,28]. It showed downregulation of auxin homeostasis and catabolic process in Figure 6A,C and Figure S7 which would make sense owing to its role in the growth and the accelerated emergence of the root during germination. Interestingly, glucosinolate metabolism is a modulator of auxin homeostasis, so this could be another explanation for changes, particularly with auxin (Figure 7A,D and Figures S2A, S3A and S7A) [60], which could still be indirectly in line with the hypothesis of hormone modulation as a possible mechanism of action.

### 3.3. Transcriptomic Plant Response to Plasma Treatment and Its Limitations

**Figure 9:**
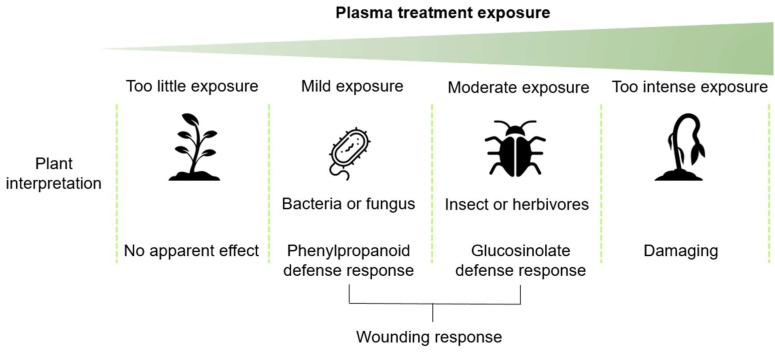
A tentative hypothesis summarizing the findings in this study where 60 s in our study was considered as mild plasma exposure and 80 s as moderate plasma exposure and resulted in phenylpropanoid or glucosinolate biosynthesis, respectively.

In this study, we demonstrated that stress and defense pathways are upregulated after plasma treatment. This seems to depend on the plasma treatment time exposure, and therefore, this interpretation is schematically proposed in Figure 9. The plasma treatment could be perceived as a wounding, perhaps from ion bombardment, even though this is not likely during indirect plasma treatment.

Plasma is a type of stress that the plant has not been previously exposed to, let alone developed a specific stress or defense response, and could thus be considered xenobiotic. Moreover, plants and pathogens continuously co-evolve where the plant will recognize pathogen-associated molecular patterns (PAMPs) or damage-associated molecular patterns (DAMPs). Plasma might not exactly mimic a bacterial or fungal infection or insect wounding; however, there might be sufficient overlap to elicit these responses, partly due to the individual plasma components that are already familiar to the plant.

More transcriptomic studies are required to validate this hypothesis; however, results are highly dependent on the conditions, for example, on the day of extraction and the tissue type. As an example, a study plasma treating *Andrographis* included three time points with the highest number of DEGs at 28 h, followed by 48 and 0 h. Future studies will include multiple time points closer to the plasma treatment as well as weeks after plasma exposure on hydrated seeds, dehydrated seeds, and seedlings. Additionally, a time series experiment targeting specific genes in the phenylpropanoid and glucosinolate pathways using qPCR will be explored. The more transcriptomic studies become available due to increased affordability, the more possibility there will be to compare the type of response that can be activated, regardless of the plasma treatment setup, as a first proof-of-concept. The next task would be understanding the operating parameters and which plasma-seed treatment is needed to trigger specific plant responses while proving that the results are reproducible, reliable, and robust for practical, industrial applications

Different responses may be elicited when using a volume DBD direct treatment since the seeds are in contact with electric fields and electron/ion bombardment compared to an indirect treatment. Additionally, depending on the seed type, it may not be possible to understand what is happening if the entire genome is not available as it is for *A. thaliana*. Although *A. thaliana* belongs to the *Brassica* family, which includes several crop species, in terms of practical applications, it would be more useful to check this on crop species to understand the applicability of this technology. Furthermore, germination rate is often selected as a parameter to judge the positive effect of plasma treatment, but it is not necessarily the standard that all should go by since effects can be observed later in time, even if absent earlier on. Koga and others found that harvest mass showed greater improvement compared to the germination rate [41]. Another author found that initial negative effects on germination were, in fact, positive over the long term for growth [61]. Therefore, other phenotypic changes should be explored, and both positive and negative results should be considered to better understand how the gene expression profiles align with the macroscopic changes. As mentioned in other papers, results in the plasma agriculture studies are more safely interpreted using multiple diagnostics in a single study to avoid misinterpretation. As an example, in the case of transcriptomics, it is generally known that mRNA levels do not necessarily correlate with protein levels [38]. Moreover, for transcript downregulation, it is not clear whether the transcripts are actively degraded, or the proteins are not replenished by the cell. Therefore, it would be useful to complement these studies with proteomics and metabolomics.

Additionally, having a molecular marker gene set for plasma exposure would be useful to know whether a plasma treatment was effective since it is difficult to know whether the plasma or the plasma-seed treatment needs to be optimized, or simply that the seed is unresponsive. This may be difficult to capture since there is great diversity in the plasma chemistry with the myriad of plasma devices, feed gas compositions, and treatment styles. Using GENEVESTIGATOR, both upregulated and downregulated gene signature profiles for 80 s were compared against other transcriptomic studies using *A. thaliana*; however, there were no clear matches between plasma and other perturbation studies (data not shown). Therefore, it remains difficult to assign a plasma response to an already known stress with our findings in this study as it seems multiple stresses can be activated. This concept of a plasma treatment gene signature would be interesting to follow up on by others as an alternative to monitoring macroscopic changes, where an effect could be present without it necessarily being expressed phenotypically.

Finally, it is always a question for living organisms of how to best use their resources since there is a trade-off between growth and defense. It needs to be stated that the application of plasma will largely depend on the context. In well-controlled environments, one might want to use plasma to harvest secondary metabolites for pharmaceutical applications, and therefore, there is little risk of abiotic and biotic stresses. Care should be taken to not jump ahead with applications since most results are in the context of a lab and are performed on a short time scale. It is unknown whether this would be advantageous or disadvantageous for the plant over the long term and in a more complex environment. The best way to evaluate this would be in a field study with stresses related to weather change, microbes, various soil conditions, and pests. Therefore, work still needs to be performed to test the feasibility of upscaling these results, although others have shown that these effects can persist for several years within the same generation on non-thermal plasma-responsive seeds [62,63].

### 3.4. Conclusions

Our findings are among the first that have performed RNA-seq on plasma-treated *A. thaliana* seeds. Here, we demonstrate that a brief (60 or 80 s) plasma treatment of dry seeds causes modifications in primary and secondary metabolisms measured after 6 days, which is evidence of a long-term memory effect. Specifically, a 60 s plasma treatment time upregulates the phenylpropanoid pathway where the seedling reinforces its cell wall with lignin and launches antimicrobial compounds such as phytoalexins, a defense response to bacteria or fungal plant pathogens. A longer plasma treatment of 80 s upregulates the glucosinolate pathway, a defense response to insects and herbivores to deter feeding. In both cases, it appears that plasma clearly acts on the plant to change the redox state and also seems to elicit a wound response. It should not be mistaken that plasma is recognized exactly as these stressors since seeds have never previously been exposed to plasma in their natural environment and therefore, plasma is still likely recognized as a foreign and abnormal stress. Indeed, accelerated germination and increased stress and defense response were all observed, although it should be underlined that this needs to be considered carefully for future applications since there is often a trade-off between growth and stress/disease resistance. Future studies should perform a time series of RNA-seq analyses after the plasma, explore the possibility of a gene signature profile specific to plasma, and include field studies where abiotic and biotic stresses are tested to check the survival of these plants under realistic conditions.

## 4. Materials and Methods

### 4.1. Seed Material

*A. thaliana* Col-0 seeds were cultivated in a plant chamber room and harvested in May 2019 using seeds from the Department of Plant Molecular Biology at the University of Lausanne. Dry seeds were 18–20 months old at the time of experiments. Seeds were stored in Eppendorf or Falcon tubes and kept at room temperature in the dark until used.

### 4.2. Germination Rate Measurements

Seeds were not sterilized nor subjected to seed preselection before plasma treatment. After plasma treatment, 30 seeds were sown immediately, or a few hours after (within the same day at the latest), on water agar plates (20 g/L, using distilled water, pH of approximately 6.7) and kept in a phytotron (AR-36L2 PlantClimatics GmbH) under continuous light using Osram L 18W 77 G13 Fluora with a 24 h light cycle at 23 °C and 65% humidity. Germination was recorded at 48 h, and seeds with roots were counted by eye. Germination rate was calculated as the number of seeds with roots divided by the total number of seeds and converted into a percentage.

### 4.3. Surface Dielectric Barrier Discharge Description

**Figure 10:**
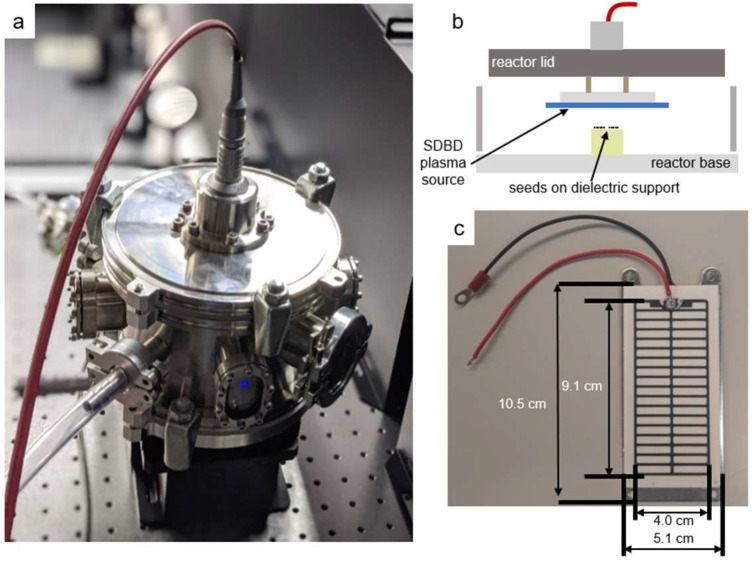
Surface dielectric barrier discharge (SDBD) plasma source enclosed in the plasma-seed treatment reactor. (**a**) Stainless steel reactor with high-voltage coaxial cable connection; (**b**) schematic of the interior with the inverted SDBD positioned above the seed substrate; (**c**) photograph of the high-voltage stripe SDBD electrode printed on an alumina dielectric plate. The ground electrode is an aluminum plate behind the dielectric.

Figure 10a shows the stainless steel reactor chamber, 18 cm diameter and 11 cm high, used to confine the SDBD air plasma and its gaseous products. The *A. thaliana* seeds were placed on Teflon cylinders, 3.7 mm below the SDBD plasma, as shown in Figure 10b. The SDBD device (Sihon Electronics) comprises an alumina dielectric and high-voltage printed electrode in a stripe pattern, as shown in Figure 10c. Additional details can be found in a previous study [29].

### 4.4. Plasma Parameters for the Seed Treatment

The operating parameters were 1 min flow flushing before treatment; 10 kHz; 8 kVpp; 60 s or 80 s plasma treatment time; 3.7 mm distance between seeds and plasma; and 2 L/min of dry synthetic air (80:20 N_2_:O_2_) controlled by mass flow controllers (Bronkhorst, Ruurlo, The Netherlands). The reactor total volume was 2.8 L with an internal gas volume of about 1.0 L. For the flow rate of 2 L/min, the gas residence time was therefore 30 s.

The source voltage waveform for all excitation frequencies was a burst of 2 sinewave cycles with a 500 Hz on/off power modulation, provided by a Rigol DG4102 signal generator amplified by a Matsusada AMPS-20B20-LC(5m) power supply. At 10 kHz sinewave frequency, 2 cycles modulated at 500 Hz corresponds to a 10% duty cycle. Seeds were placed on Teflon spacers during treatment or on ceramic plates to reduce the seed-plasma gap.

Humidity was measured using a Vaisala model HM42 probe and ranged between 1.5% and 3% RH. This low humidity is consistent with the use of dry synthetic air and only small out-gassing of humidity from the reactor walls. The temperature was measured at the center of the SDBD with a FLIR E85 infrared camera, and the measurements for 60 and 80 s were 31.2 and 31.8 °C, respectively, using a 10% duty cycle [29].

### 4.5. Germination Statistics

Differences in germination rate between the control and plasma-treated samples were assessed using ordinary one-way ANOVA. Each treatment group was compared to their respective control, and the bar graph in Figure 1 represents two independent experiments with 3 replicates each for a total of 6 replicates. GraphPad Prism 9 (GraphPad Software, Inc., San Diego, CA, USA) was used for statistical analyses. All *p*-values < 0.05 were considered to be significant and given directly in Figure 1.

### 4.6. RNA Isolation, Library Construction, and RNA Sequencing

The seeds were treated with plasma and planted on agar shortly after within hours. The germination rate was measured 48 h after sowing and was grown for another 4 days until RNA was extracted (6 days after sowing). Total RNA was extracted from three biological replicates obtained from 6-day-old seedlings (up to 100 mg) using Precellys (Bertin, Montingy-le-Bretonneux, France) and lysing kit with 1.4 mm zirconium beads in 0.5 mL tubes. The settings used were 6000 rpm for 30 s, followed by a 10 s break, and finished with 6000 rpm for 30 s, all performed at 4 °C. InnuPREP Plant RNA kit (Analytic Jena, Jena, Germany) was used for RNA isolation and quantified with nanodrop (DS-11 Microvolume Spectrophotometer).

RNA quality was assessed on a Fragment Analyzer (Agilent Technologies, Santa Clara, CA, USA), and all RNAs had an RNA quality number (RQN) above 7.9. Library preparation and RNA-seq were performed at the Lausanne Genomic Technologies Facility, University of Lausanne, Switzerland (https://www.unil.ch/gtf (accessed on 15 March 2021)). RNA-seq libraries were prepared from 400 ng of total RNA with the Illumina TruSeq Stranded mRNA reagents (Illumina, San Diego, CA, USA) using a unique dual indexing strategy and following the official protocol automated on a Sciclone liquid handling robot (PerkinElmer, Waltham, MA, USA). Libraries were quantified by a fluorimetric method (QubIT, Life Technologies, Carlsbad, CA, USA), and their quality was assessed on a Fragment Analyzer (Agilent Technologies).

Cluster generation was performed with 2 nM of an equimolar pool from the resulting libraries using the Illumina HiSeq 3000/4000 SR Cluster Kit reagents, then sequenced on the Illumina HiSeq 4000 SR platform (single end) using HiSeq 3000/4000 SBS Kit reagents for 150 cycles (single end). Sequencing data were demultiplexed using the bcl2fastq2 Conversion Software (version 2.20, Illumina). The analysis resulted in approximately 31–37 million of 150 bp long single-end reads for each library independently (Table S1).

After sequencing, raw reads were subjected to quality control (phred score > 20) and adapter trimming using FastQC (0.11.976) and BBDuk. Reads matching ribosomal RNA sequences were removed with fastq_screen (v. 0.9.3). Reads were aligned against the Arabidopsis reference genome sequence (Araport11) using STAR v2.7.5 using default parameters (Dobin et al., 2013). FeatureCounts v1.6.2 was used to generate the count matrix and to calculate gene expression values as raw read counts. RPKM was obtained from FeatureCounts (in house script) to make heatmaps. Count read values were analyzed using the DESeq2 package from R software v1.30.1 [33] after rlog transformation to identify the differentially expressed genes (DEGs). A *t*-test was performed to identify differential enrichment between control and treated samples. To identify GO categories of differentially expressed genes, ShinyGO v.0.66 software was used [34]. The results were based on customized background genes from our RNA-seq, which yield more accurate results for enrichment analysis [40]. The transcriptome data are available in NCBI Bioproject Code: PRJNA800224.

## Data Availability

The data presented in this study are available in this article, supplementary material and the transcriptome data are available in NCBI Bioproject Code: PRJNA800224.

## Supplementary Materials

The following supporting information can be downloaded at: https://www.mdpi.com/article/10.3390/ijms23063070/s1.
