# Supplementary material for: RNA Sequencing of Arabidopsis thaliana Seedlings after Non-Thermal Plasma-Seed Treatment Reveals Upregulation in Plant Stress and Defense Pathways
Source: Int J Mol Sci. 2022 Mar 12;23(6):3070. doi: 10.3390/ijms23063070 (PMC8955755; doi:10.3390/ijms23063070)
Supplement: Supplementary file 1 [file ijms-23-03070-s001.zip › ijms-1608280-supplementary.pdf]

International Journal of
Molecular Sciences
MDPI

Supplemental materials

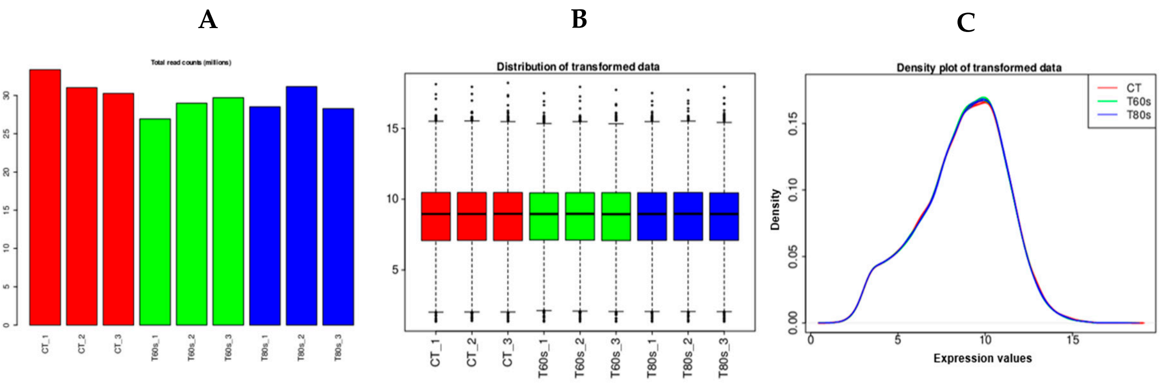

Figure S1. Quality control for control, 60 s and 80 s samples. (A) Genes retained in each sample (B) normalization of samples (C) density plot to demonstrate that profiles are similar to proceed with analysis.

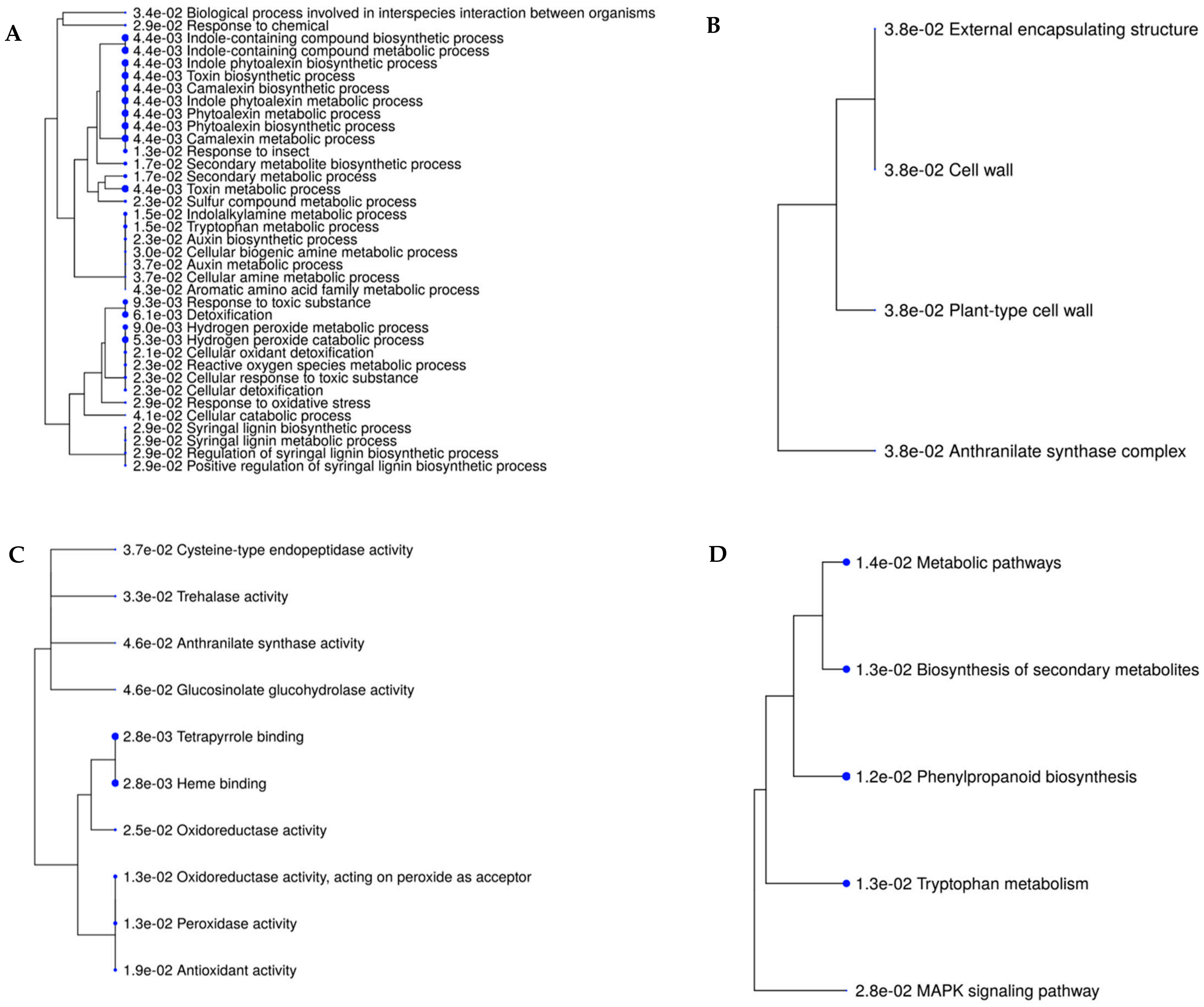

Figure S2. A hierarchical clustering tree for the upregulated genes after 60 s plasma treatment, summarizing the correlation among significant pathways within GO categories (A) biological process (B) cellular component (C) molecular function (D) KEGG pathway. Pathways with many shared genes are clustered together. Bigger dots indicate more significant P-values.

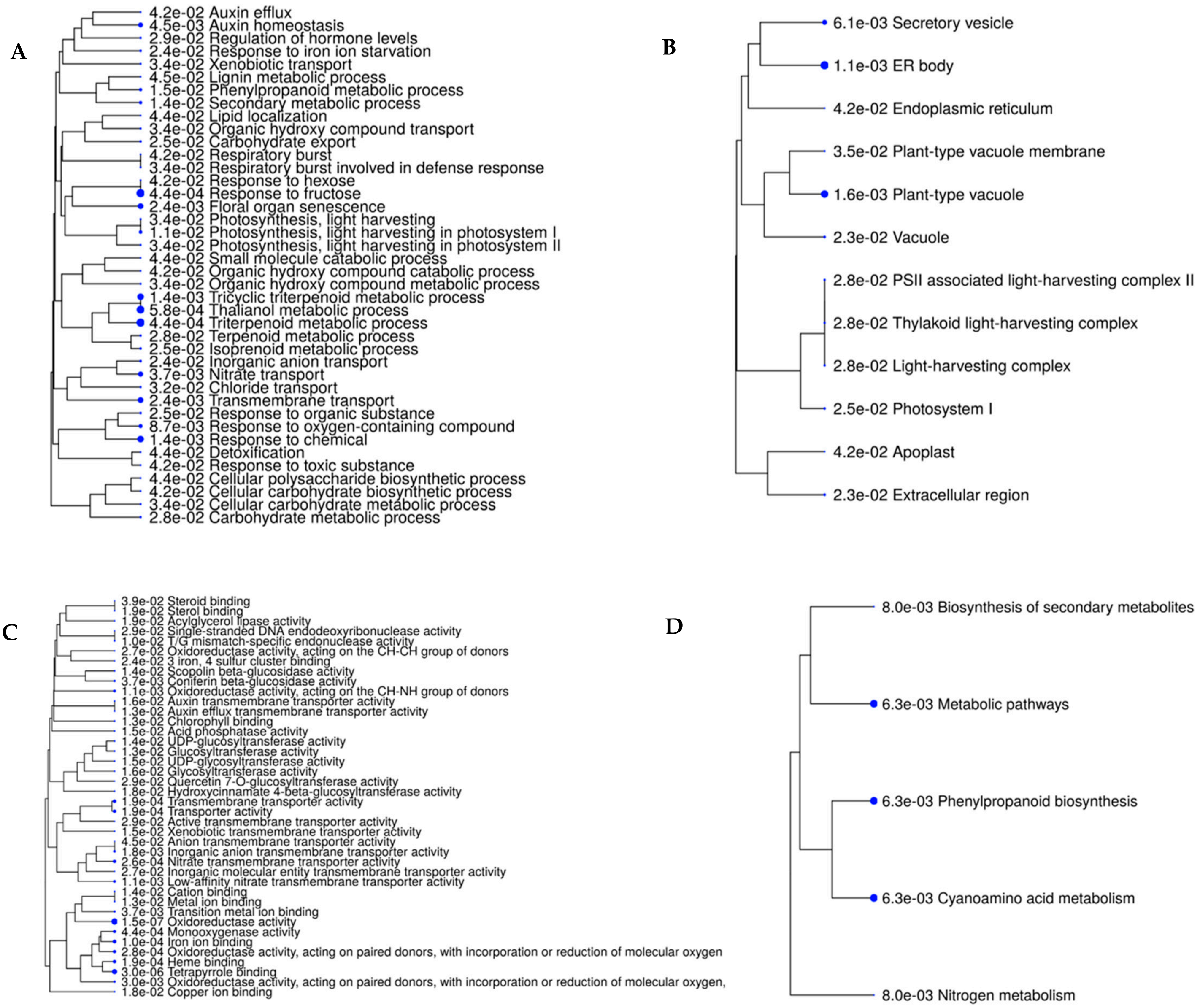

Figure S3. A hierarchical clustering tree for the downregulated genes after 60 s plasma treatment, summarizing the correlation among significant pathways within GO categories (A) biological process (B) cellular component (C) molecular function (D) KEGG pathway. Pathways with many shared genes are clustered together. Bigger dots indicate more significant P-values.

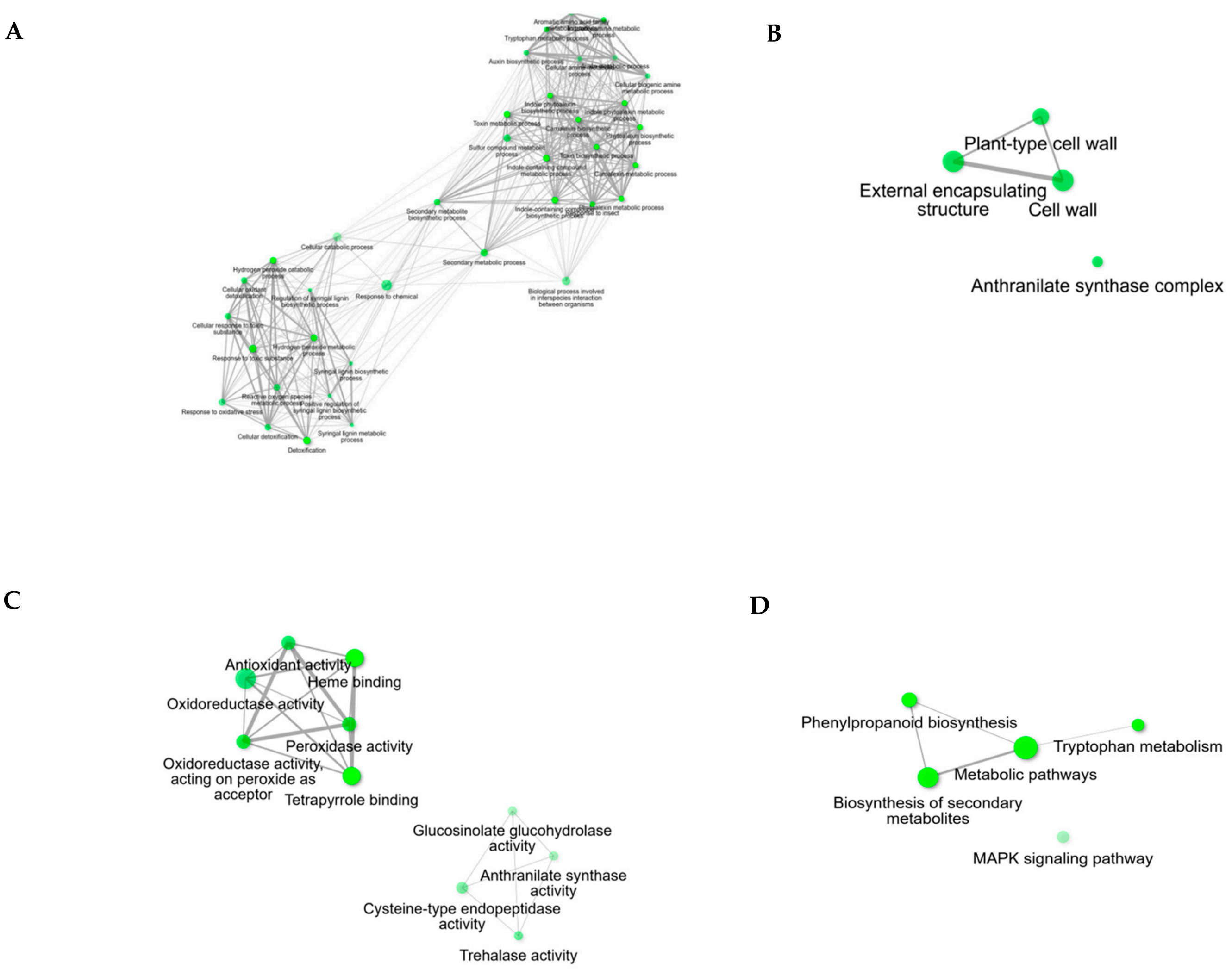

Figure S4. Gene upregulation after 60 s plasma treatment. Network maps show the relationship between enriched pathways in plasma-treated seedlings. From top to bottom, GO categories are in order: biological process, cellular component, molecular function, and KEGG pathway. Two pathways (nodes) are connected if they share 20% or more genes. Darker nodes are more significantly enriched gene sets. Bigger nodes represent larger gene sets. Thicker edges represent more overlapped genes.

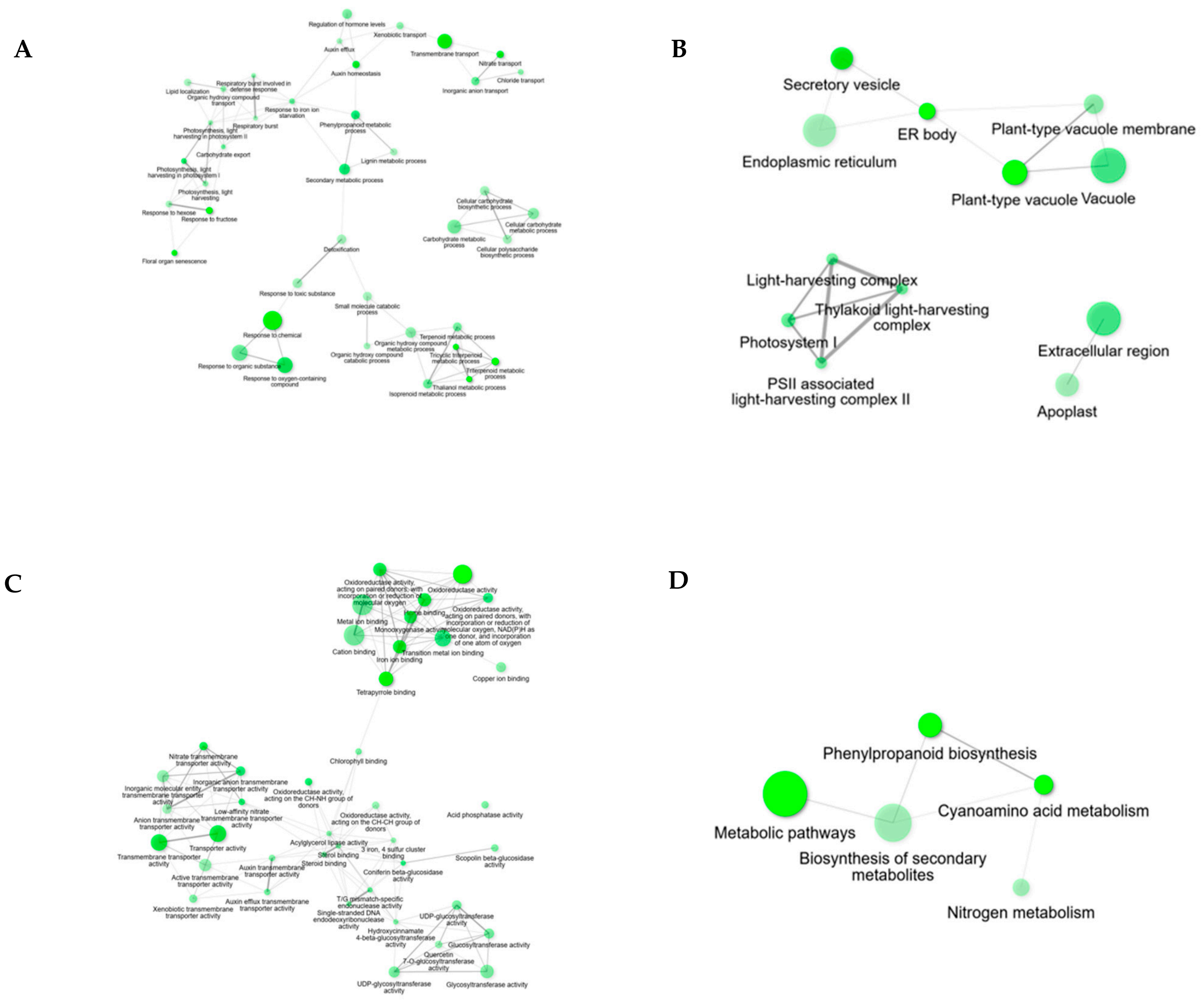

Figure S5. Gene downregulation after 60 s plasma treatment. Network maps show the relationship between enriched pathways in plasma-treated seedlings. From top to bottom, GO categories are in order: biological process, cellular component, molecular function, and KEGG pathway. Two pathways (nodes) are connected if they share 20% or more genes. Darker nodes are more significantly enriched gene sets. Bigger nodes represent larger gene sets. Thicker edges represent more overlapped genes.

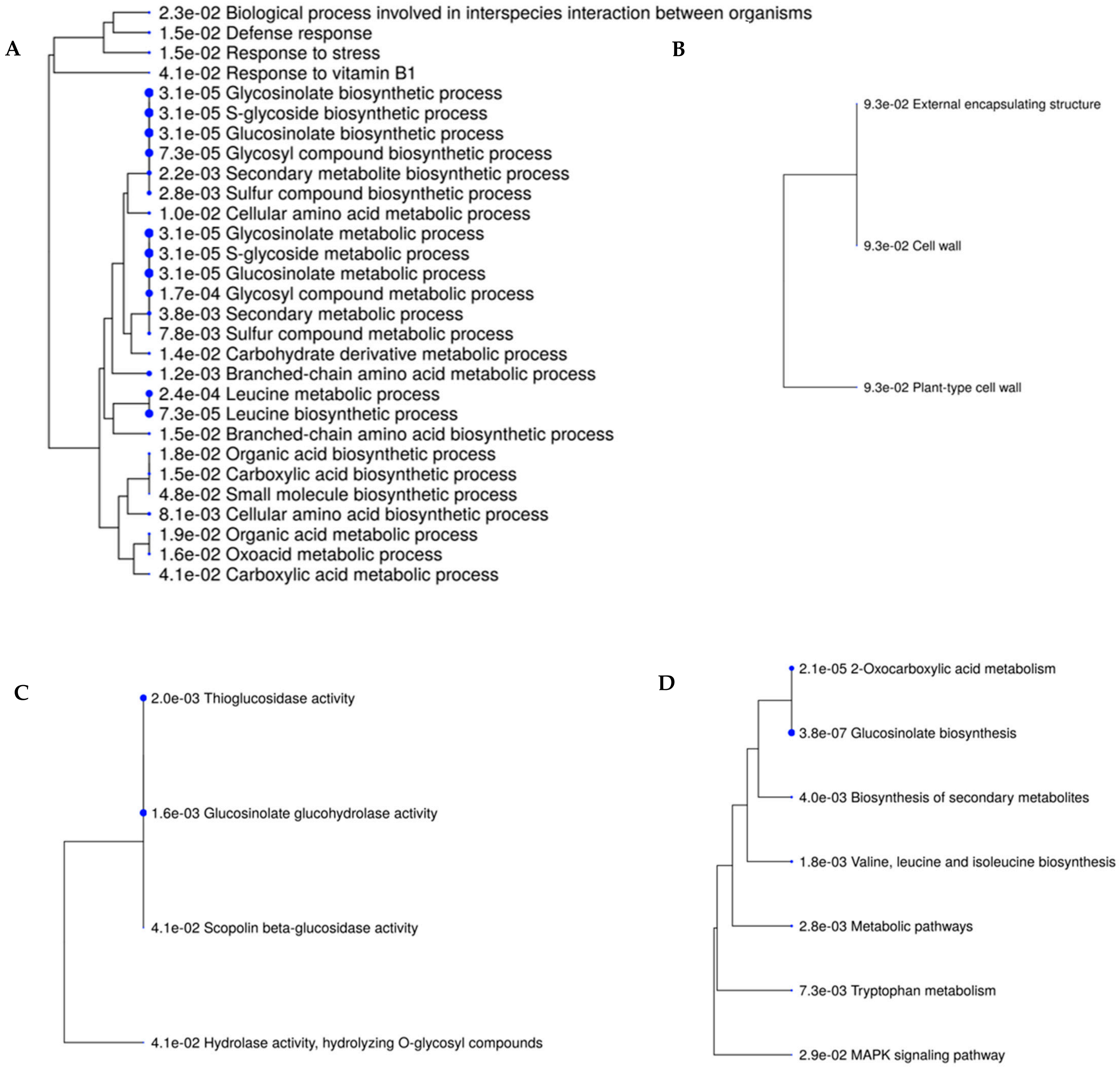

Figure S6. A hierarchical clustering tree for the upregulated genes after 80 s plasma treatment, summarizing the correlation among significant pathways within GO categories (A) biological process (B) cellular component (C) molecular function (D) KEGG pathway. Pathways with many shared genes are clustered together. Bigger dots indicate more significant P-values.

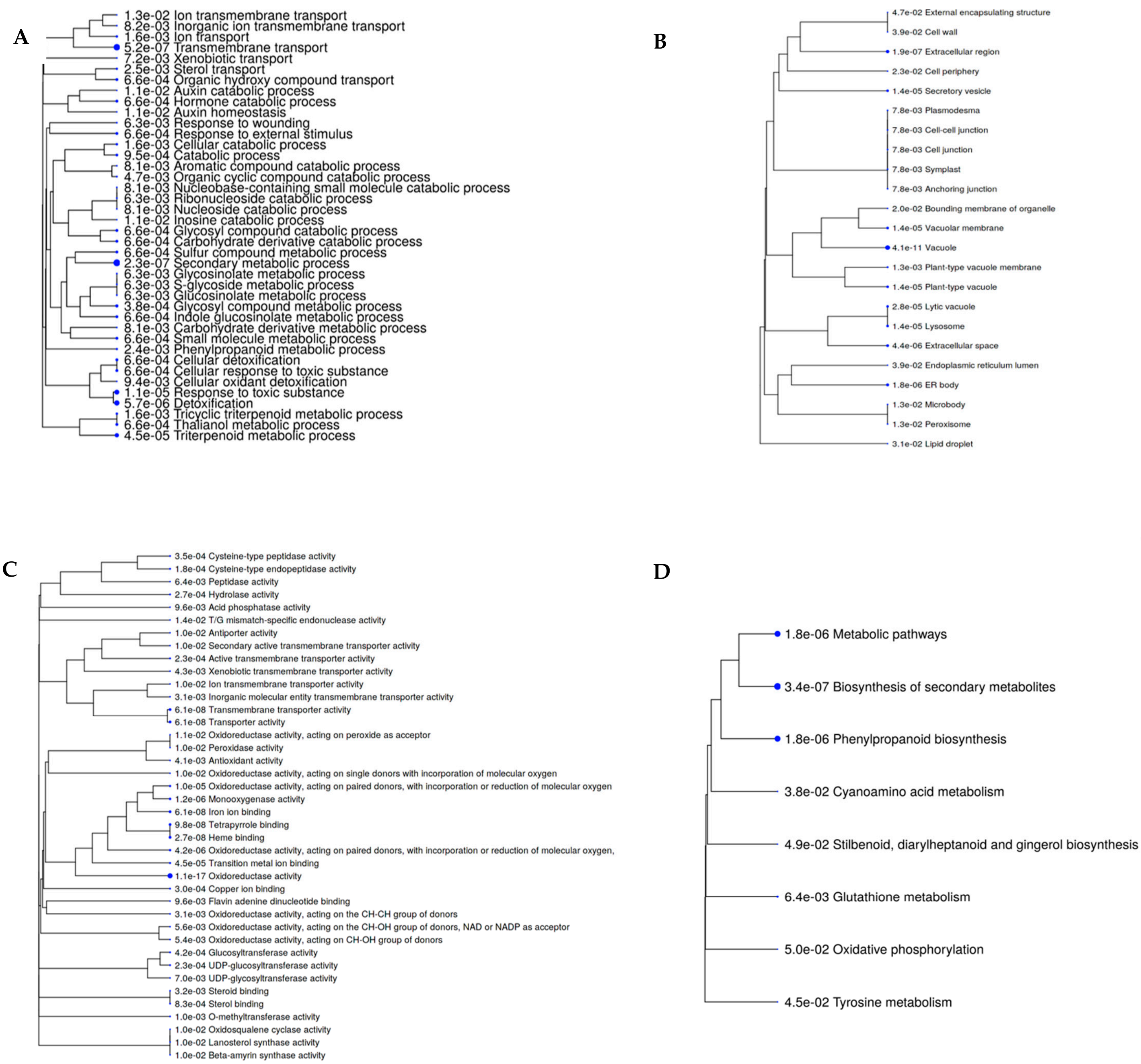

Figure S7. A hierarchical clustering tree for the downregulated genes after 80 s plasma treatment, summarizing the correlation among significant pathways within GO categories (A) biological process (B) cellular component (C) molecular function (D) KEGG pathway. Pathways with many shared genes are clustered together. Bigger dots indicate more significant P-values.

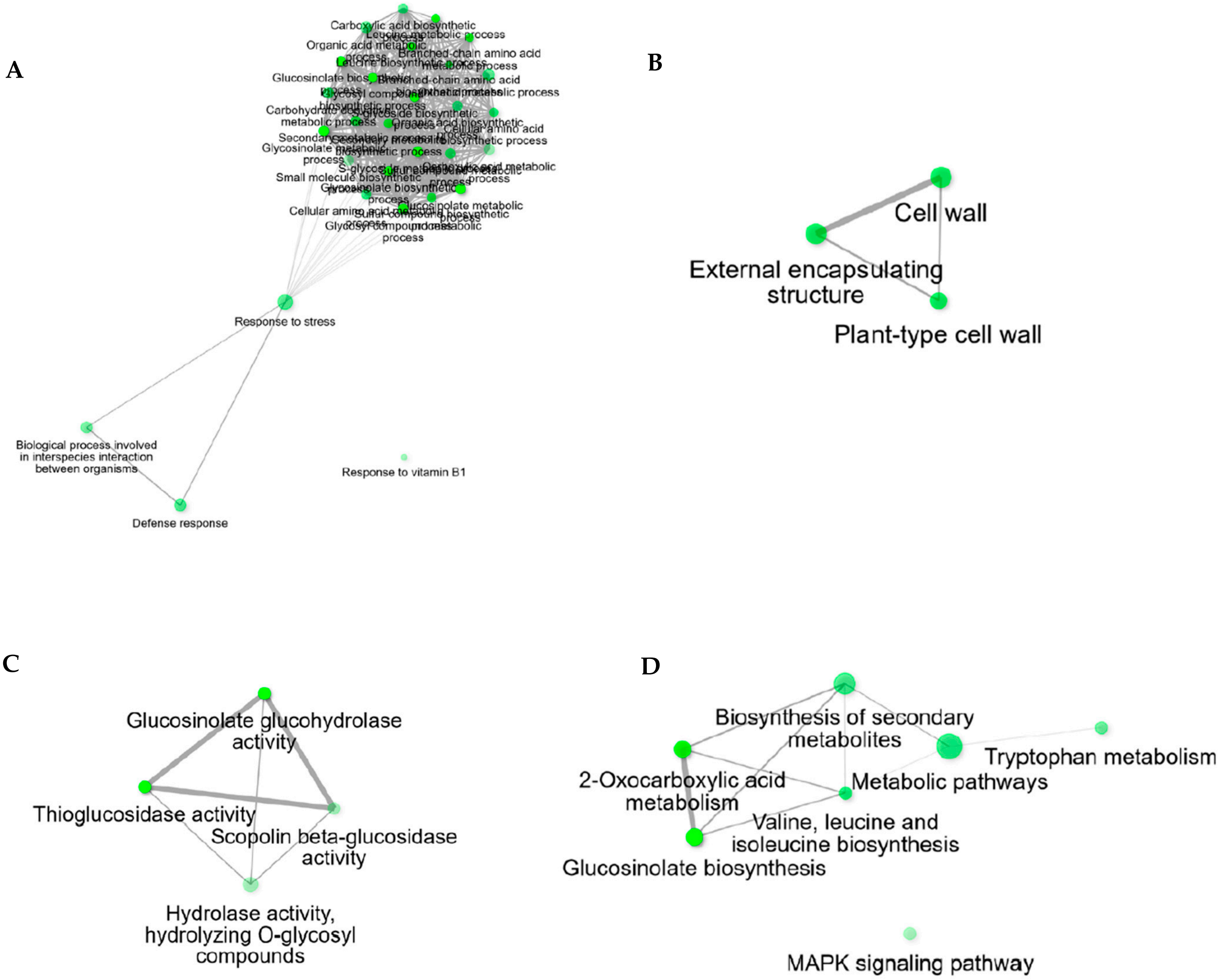

Figure S8. Gene upregulation after 80 s plasma treatment. From top to bottom, GO categories are in the following order: (A) biological process, (B) cellular component, (C) molecular function, and (D) KEGG pathway. Network maps show the relationship between enriched pathways in plasma-treated seedlings. Two pathways (nodes) are connected if they share 20% or more genes. Darker nodes are more significantly enriched gene sets. Bigger nodes represent larger gene sets. Thicker edges represent more overlapped genes.

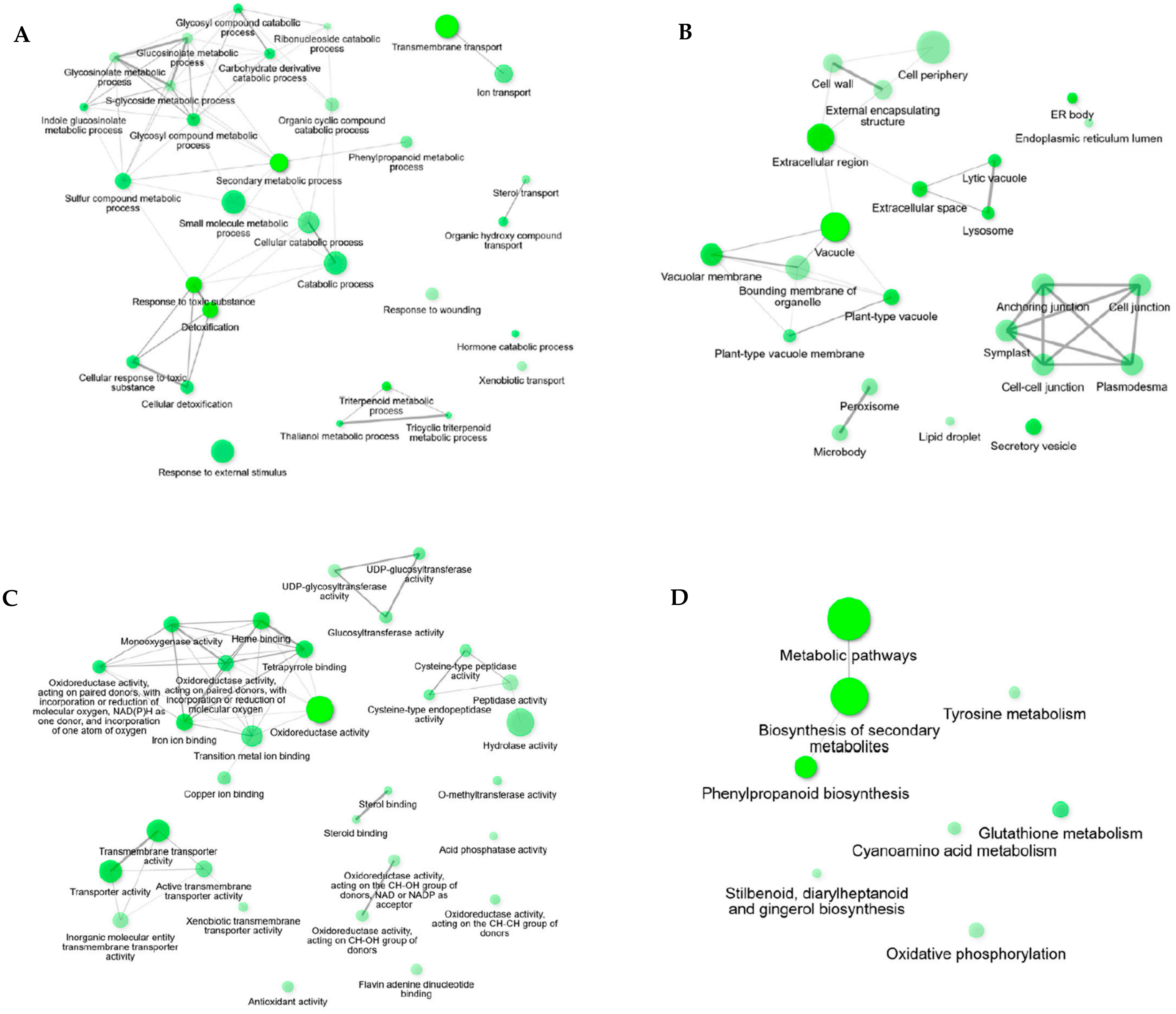

Figure S9. Gene downregulation after 80 s plasma treatment. From top to bottom, GO categories are in the following order: (A) biological process, (B) cellular component, (C) molecular function, and (D) KEGG pathway. Network maps show the relationship between enriched pathways in plasma-treated seedlings. Two pathways (nodes) are connected if they share 20% or more genes. Darker nodes are more significantly enriched gene sets. Bigger nodes represent larger gene sets. Thicker edges represent more overlapped genes.

Table S1. Number of NGS-RNA-seq reads before and after quality check on the raw sequencing data for 60 s and 80 s

Sample name	Raw reads	Trimmed reads	Mean GC	Condition
control 1	37031768	34794479	45	control
control 2	33884449	32507959	45	control
control 3	33288964	31830410	45	control
60 s 1	29560591	28386155	45	60 s
60 s 2	31755491	30348719	45	60 s
60 s 3	32244438	31058363	45	60 s
80 s 1	31068712	29937596	45	80 s
80 s 2	33745593	32428165	45	80 s
80 s 3	31108401	29729217	45	80 s

Table S2. Number of clean reads mapped against *A. thaliana* genome for 60 s and 80 s

Sample	Input reads	Unique	Multi	Unmapped	Mismatch ratio	Assigned GTF
control 1	34794479	33792317 (97.1198%)	555033 (1.59518%)	447129 (1.28506%)	0.06%	33379423 (95.9331%)
control 2	32507959	31460285 (96.7772%)	672683 (2.06929%)	374991 (1.15354%)	0.06%	31023336 (95.433%)
control 3	31830410	30686662 (96.4067%)	681168 (2.13999%)	462580 (1.45326%)	0.06%	30273545 (95.1089%)
60 s 1	28386155	27435554 (96.6512%)	679223 (2.3928%)	271378 (0.956022%)	0.06%	26931867 (94.8768%)
60 s 2	30348719	29391853 (96.8471%)	567223 (1.86902%)	389643 (1.28389%)	0.06%	29009927 (95.5886%)
60 s 3	31058363	30157945 (97.1009%)	587937 (1.89301%)	312481 (1.00611%)	0.06%	29726503 (95.7118%)
80 s 1	29937596	28988426 (96.8295%)	662731 (2.21371%)	286439 (0.956787%)	0.06%	28514100 (95.2451%)
80 s 2	32428165	31571882 (97.3594%)	522820 (1.61224%)	333463 (1.02831%)	0.06%	31166918 (96.1106%)
80 s 3	29729217	28722931 (96.6152%)	627181 (2.10965%)	379105 (1.27519%)	0.06%	28292247 (95.1665%)

Table S3. Comparison with Cui et al. 2021 paper

Variable	Cui et al. paper	Waskow et al. paper
Plasma device	Hexagonal mesh SDBD	Grid of stripes SDBD
Plasma device materials	Copper and FR4	Metal paste on alumina ceramic
Plasma-seed gap distance	5 mm	3.7 mm
Feed gas	Ambient air<br>No flow given<br>RH 65%	Synthetic air (80:20 N_2:O_2)<br>2 L/min controlled flow<br>RH 2%
Operating parameters	8 kHz, 5 kV<br>2.5 W consumption<br>0.055 W/cm^2	10 kHz, 8 kV,<br>2-3 W consumption,<br>0.03 - 0.08 W/cm^2
Power supply	Continuous sinewave	AC 10% duty cycle<br>modulated at 500 Hz
Temperature	28.5°C	31.8°C
Seed storage and handling	vernalized and stored with silica gel,<br>sterilized seeds,<br>used ½ MS agar plates	stored in a locked Eppendorf tube<br>dry conditions at room temperature,<br>not sterilized,<br>used water agar
Plasma treatment procedure	grew seedlings until 4 days,<br>transplanted to water,<br>plasma treated,<br>planted on ½ MS agar	plasma treated dry seeds,<br>planted on water agar
Seed sample size	35 seeds	30 seeds
Light cycle	23°C, 16 h light	23°C, continuous light
Plasma treatment time	60 s	60 s and 80 s
RNA extraction	48 hours after plasma treatment<br>extracted from 6-day-old seedlings	144 hours after plasma treatment<br>extracted from 6-day-old seedlings
